# Reappraisal of the diagnostic and prognostic value of morning stiffness in arthralgia and early arthritis: results from the Groningen EA*R*C, Leiden EA*R*C, ESPOIR, Leiden EAC and REACH

**DOI:** 10.1186/s13075-015-0616-3

**Published:** 2015-04-23

**Authors:** Jessica AB van Nies, Celina Alves, Audrey LS Radix-Bloemen, Cécile Gaujoux-Viala, Tom WJ Huizinga, Johanna MW Hazes, Elisabeth Brouwer, Bruno Fautrel, Annette HM van der Helm-van Mil

**Affiliations:** Department of Rheumatology, Leiden University Medical Center, Albinusdreef 2, 2333 ZC Leiden, the Netherlands; Department of Rheumatology, Erasmus MC, University Medical Center Rotterdam, ‘s-Gravendijkwal 230, 3015 CE Rotterdam, the Netherlands; Department of Rheumatology and Clinical Immunology, University of Groningen, University Medical Center, Antonius Deusinglaan 1, 9713 AV Groningen, the Netherlands; Department of Rheumatology, Nîmes University Hospital; EA 2415, Montpellier I University, 4 Rue du Professeur Robert Debré, 30029 Nîmes, France; Department of Rheumatology, Université Pierre et Marie Curie Curie – Paris 6, GRC08, Institut Pierre Louis de d’Epidémiologie et Santé Publique, Pitie-Salpetriere Hospital, 47-83 Boulevard de l’Hôpital, 75013 Paris, France

## Abstract

**Introduction:**

Morning stiffness is assessed daily in the diagnostic process of arthralgia and arthritis, but large-scale studies on the discriminative ability are absent. This study explored the diagnostic value of morning stiffness in 5,202 arthralgia and arthritis patients and the prognostic value in early rheumatoid arthritis (RA).

**Methods:**

In arthralgia patients referred to the Early Arthritis *Recognition* Clinics (EA*R*C) of Leiden (n = 807) and Groningen (n = 481) or included in the Rotterdam Early Arthritis Cohort (REACH) study (n = 353), the associations (cross-sectional analyses) between morning stiffness and presence of arthritis at physical examination were studied. In early arthritis patients, included in the Leiden Early Arthritis Clinic (EAC) (n = 2,748) and Evaluation et Suivi de POlyarthrites Indifférenciées Récentes (ESPOIR) (n = 813), associations with fulfilling the 2010-RA criteria after one year were assessed. In 2010-RA patients included in the EAC (n = 1,140) and ESPOIR (n = 677), association with the long-term outcomes of disease-modifying antirheumatic drug (DMARD)-free sustained remission and radiological progression were determined. Morning stiffness was defined as a duration ≥60 minutes; sensitivity analyses were performed for other definitions.

**Results:**

In arthralgia, morning stiffness (≥60 minutes) associated with the presence of arthritis; Leiden EA*R*C odds ratio (OR) 1.49 (95% CI 1.001 to 2.20), Groningen EA*R*C OR 2.21 (1.33 to 3.69) and REACH OR 1.55 (0.97 to 2.47) but the areas under the receiver operating characteristic curve (AUCs) were low (0.52, 0.57, 0.54). In early arthritis, morning stiffness was associated with 2010-RA independent of other predictors (Leiden EAC OR 1.72 (95% CI 1.31 to 2.25, AUC 0.68), ESPOIR OR 1.68 (1.03 to 2.74, AUC 0.64)). Duration of ≥30 minutes provided optimal discrimination for RA in early arthritis. Morning stiffness was not associated with radiological progression or DMARD-free sustained remission.

**Conclusions:**

Morning stiffness in arthralgia and early arthritis is associated with arthritis and RA respectively. This supports the incorporation of morning stiffness in the diagnostic process.

**Electronic supplementary material:**

The online version of this article (doi:10.1186/s13075-015-0616-3) contains supplementary material, which is available to authorized users.

## Introduction

Morning stiffness is common in patients with rheumatoid arthritis (RA); it affects the ability to function in the morning [1], the quality of life and is associated with work loss [2]. Presence of morning stiffness, together with fatigue, are often mentioned as one of the first symptoms of RA. Therefore, morning stiffness is usually assessed in the diagnostic process of patients presenting with arthralgia or arthritis [3].

The scientific data on the diagnostic value of this symptom are surprisingly scanty. In the literature, it has been mentioned that morning stiffness is a poor discriminator between RA and other rheumatologic disorders [4,5]. However, these conclusions are predominantly based on two studies, with relatively small sample sizes. The first study compared 93 RA patients and 46 patients with non-inflammatory joint diseases [4]. The second study compared 31 RA patients with 23 systemic lupus erythematosus (SLE) patients and 34 osteoarthritis (OA) patients [5]. It has also been suggested that morning stiffness is commonly present in the general population and not specific for RA [6,7]. This notion is also based on only two studies. A large study revealed a prevalence of morning stiffness of 37% when defined as stiffness of ≥15 minutes; this definition is generally not considered as typical for RA [6]. The other study originated from the early 1950s and reported morning stiffness in 19% of persons without RA, but a definition of morning stiffness was not provided [7]. Altogether, there is not much evidence on the diagnostic value of morning stiffness. It is also not part of the 2010 American College of Rheumatology (ACR)/European League Against Rheumatism (EULAR) classification criteria for RA, whereas it had been included in the 1958 American Rheumatism Association (ARA) criteria and the 1987 ACR criteria for RA [8-10]. Because of the paradox of the lack of large-scale studies focusing on morning stiffness, and the use of morning stiffness in daily practice by rheumatologists and general practitioners, we set out to study the diagnostic value of morning stiffness in arthralgia and early arthritis by studying different European datasets and cohorts. The basic aim to this study was to evaluate the diagnostic value of morning stiffness. Because the diagnostic value is dependent on the patient population in which a test is performed, we studied the discriminative ability of morning stiffness in two situations. First, in cross-sectional analyses on patients with arthralgia, the association between morning stiffness and the presence of arthritis at physical examination was studied. This information is relevant for general practitioners (GPs) and other physicians who encounter patients with joint symptoms in their practices and who have limited experience in joint examination. Second, in early arthritis, the ability of morning stiffness to discriminate patients with RA from other early arthritis patients was assessed.

Although it is known that morning stiffness is associated with the disease activity [11], functional disability [12] and work loss [2] in RA, it is undetermined whether morning stiffness at first presentation is a risk factor for a more severe disease reflected by structural damage or disease persistence. To evaluate this, associations with radiographic progression and achieving DMARD-free sustained remission (the absence of disease persistence) were assessed in two longitudinal cohort studies.

## Methods

### Patients

All datasets and cohorts used are described in more detail elsewhere [13-16].

In short, the arthralgia patients were referred to the Early Arthritis *Recognition* Clinics (EA*R*C) from Leiden and Groningen (the Netherlands) or included in the Rotterdam Early Arthritis Cohort (REACH) (the Netherlands). The EA*R*Cs were initiated to reduce referral delay by GPs. It was observed that the GP delay contributed to two-thirds of the total delay between symptom onset and first visit to a rheumatologist in the Netherlands and that GPs frequently applied a ‘wait-and-see’ approach if they were unsure of the presence of arthritis. Therefore, GPs were instructed to refer to the EA*R*C if they were undecided. The EA*R*Cs are early access clinics in which experienced rheumatologists screen patients on the presence of arthritis by physical examination; no laboratory investigations are done [13,17]. The studied arthralgia patients visited the Leiden EA*R*C between September 2010 and August 2013 and the Groningen EA*R*C between October 2010 and January 2014. The EA*R*C is primarily part of our care. Patients visiting the EA*R*Cs were not subjected to procedures that were done for scientific purposes, such as blood taking for biobanking. Therefore, in line with the Dutch law ‘*Wet medisch-wetenschappelijk onderzoek met mensen*’ (translated as ‘the law on medical and scientific research involving people’), patients were not asked to sign an informed consent form.

The REACH study is an inception cohort that was initiated in the Rotterdam area, the Netherlands, in 2004. Inclusion required either pain or loss of movement in ≥2 joints or >1 swollen joint and ≥2 of the following items: unable to clench a fist in the morning, pain when shaking someone’s hand, pins and needles in the fingers, difficulties wearing rings or shoes, a family history of RA, morning stiffness >1 hour, unexplained fatigue, all <1 year [14]. The patients studied here were referred with joint symptoms by GPs between 2004 and 2009 and the presence of arthritis was assessed at the first visit. REACH was approved by the ethics committees of all three participating hospitals (Erasmus MC University Medical Center Rotterdam, Sint Franciscus Gasthuis Rotterdam and Maasstad Ziekenhuis Rotterdam); all patients gave written informed consent.

The early arthritis patients were included in the Leiden Early Arthritis Clinic (EAC) and the Evaluation et Suivi de POlyarthrites Indifférenciées Récentes (ESPOIR) cohort. The Leiden EAC is a population-based inception cohort that started in 1993. Inclusion required the presence of arthritis of ≥1 joint at physical examination and symptom duration <2 years [15]. The patients studied were included between 1993 and 2011. The EAC was approved by the local Leiden University Medical Center (LUMC) ethics committee, all patients gave informed consent. ESPOIR is a nationwide cohort in which 14 rheumatology centres throughout France collaborate. Early arthritis patients are included if the treating rheumatologists suspected them of having or developing RA. Further, for inclusion patients had to be aged 18 to 70 years and to have ≥2 swollen joints for >6 weeks and <6 months. Patients studied were included between 2002 and 2005 [16]. ESPOIR was approved by the ethics committee of Montpellier; all patients gave written informed consent. In both the EAC and ESPOIR questionnaires were filled in, joint counts performed and laboratory evaluations done at baseline. Patients were followed prospectively with yearly follow-up visits; these included clinical and laboratory evaluations and radiographs of hands and feet.

### Assessment of morning stiffness

In all cohorts the duration of morning stiffness was reported in minutes. In the EA*R*Cs, patients answered questionnaires on the presence and duration of morning stiffness (Additional file 1). In the EAC, ESPOIR and REACH the questions on presence and duration of morning stiffness were asked by trained research nurses (Additional file 1). Patients were not asked for specific locations of stiffness. Morning stiffness duration was dichotomized into <60 and ≥60 minutes. Sensitivity analyses were performed with ≥30 and ≥90 minutes as cutoffs. To evaluate the consistency in results when morning stiffness was assessed differently, analyses on arthritis patients were repeated with the severity of morning stiffness instead of the duration. The severity was assessed using a visual analogue scale (VAS) in 1,959 EAC patients included between 1993 and February 2010 and in all ESPOIR patients. For analyses, the VAS was divided into the three categories; mild 0 to 33 millimeter (mm), moderate 34 to 67 mm and severe 68 to 100 mm. In the arthralgia datasets the severity was not recorded. In all patients studied, morning stiffness was assessed at the first visit, when patients were not treated with disease-modifying antirheumatic drugs (DMARDs).

### Outcomes

**Figure 1:**
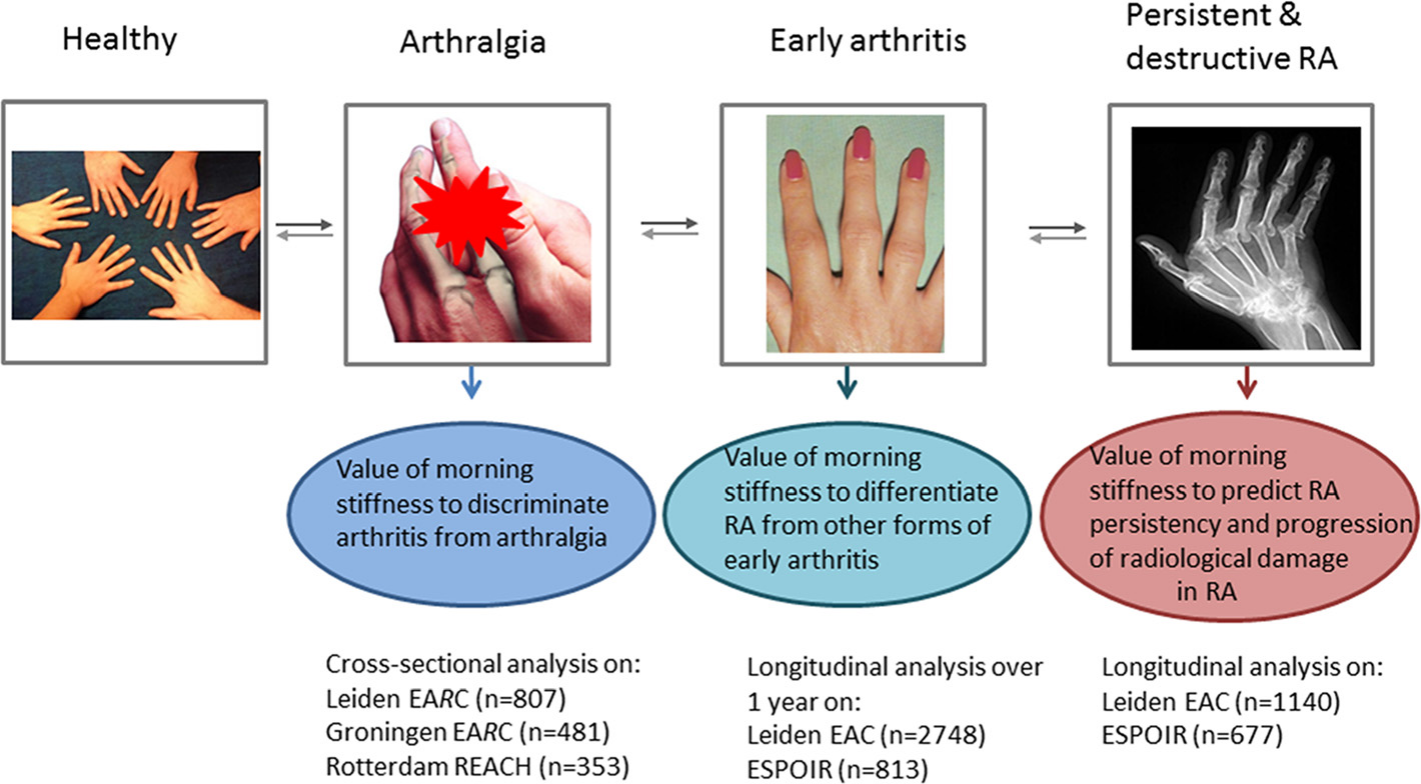
Outline of study questions. Of the 2010-RA patients in the EAC, radiographs were scored for the patients included between 1993 and 2006 (n = 636). Baseline characteristics of RA patients included before or after 2006 were not different. In ESPOIR, radiographic data was available for 659 of 677 RA patients. Here also, baseline characteristics of patients with and without radiographs were not different. EAC, Early Arthritis Clinic; ESPOIR, Evaluation et Suivi de POlyarthrites Indifférenciées Récentes; RA, rheumatoid arthritis.

The outcomes were different in the three parts of this study (Figure 1).

#### Diagnostic value in arthralgia

In arthralgia patients, the outcome was the presence of arthritis ascertained at physical examination by experienced rheumatologists (assessed at the same visit when morning stiffness was evaluated). In both EA*R*Cs, a small proportion of patients (58 and 25) had no evident arthritis but were also not classified as having ‘no arthritis’ because the rheumatologists suspected these patients of RA development; these patients were excluded from analyses.

#### Diagnostic value in early arthritis

In early arthritis, we aimed to assess the diagnostic value of morning stiffness and here the outcome was the presence of RA after one year. RA was defined as fulfilling the 2010 ACR/EULAR criteria for RA during the first year. An advantage of the 2010-RA criteria is that morning stiffness is not included, preventing circle reasoning. Since during the first weeks the diagnoses may not yet be definitive, the classification after year one was evaluated. These first two parts evaluated the diagnostic value.

#### Prognostic value within RA

Third, within 2010-RA patients, the prognostic value was assessed by studying two long-term outcomes. Structural damage was assessed using serial hands and feet radiographs that were scored according to the Sharp/van der Heijde (SHS) method with known time order and blinded to clinical data. In the EAC, radiographs were scored of patients included between 1993 and 2006. The follow-up was seven years in the EAC and three years in ESPOIR. The within-reader intraclass correlation coefficients (ICCs) were 0.91 and 0.87 for two readers in the EAC and 0.97 in ESPOIR. DMARD-free sustained remission, the opposite of disease persistence, was defined as the sustained absence of arthritis after discontinuation of DMARD therapy, including biologics and glucocorticoids (systemic and intra-articular), for the entire period of follow-up, which was at least one year [18]. In the EAC, it was assessed by exploring the medical files until 10 years of follow-up. In ESPOIR, it was assessed over five years of follow-up by reviewing the structured visits in the ESPOIR database.

### Analyses

Characteristics were compared using Student *t* tests, Mann–Whitney tests or chi-square tests when appropriate. Associations of morning stiffness in arthralgia and early arthritis were done using logistic regression analyses. All analyses were adjusted for age and gender (although morning stiffness was not correlated with age in arthralgia or early arthritis). In early arthritis further adjustments were made for anti-citrullinated protein antibody (ACPA), rheumatoid factor (RF), swollen joint count (SJC), erythrocyte sedimentation rate (ESR) and symptom duration at baseline. The test characteristics (sensitivity, specificity), positive/negative predictive values (PPV/NPV) and area under the receiver operating characteristic curve (AUC) were calculated. This curve was used to derive the morning stiffness duration with the optimal discriminative ability (Youden’s index). Associations between morning stiffness at baseline and radiographic progression were studied using multivariate normal regression analysis with log-transformed radiographic data as response variable as described elsewhere [19,20]. Analyses on DMARD-free sustained remission were done by Kaplan-Meier survival curves and Cox proportional hazard regression models with morning stiffness as an independent variable. Analyses on radiographic progression and DMARD-free sustained remission were adjusted for age, gender, ACPA and inclusion period as a proxy for differences in treatment strategy as described elsewhere [17]. SPSS version 20.0 was used (IBM Corp, Armonk, NY, USA).

## Results

### Diagnostic value of morning stiffness in arthralgia

In the Leiden EA*R*C, 807 arthralgia patients were seen, in the Groningen EA*R*C, 481 arthralgia patients and in the REACH, 353 patients were included. Arthritis was observed in 372 (46%), 267 (56%) and 181 (51%) patients respectively. Table 1 presents baseline characteristics. The median (interquartile range (IQR)) duration of morning stiffness was 10 (0 to 30), 10 (0 to 30) and 30 (0 to 60) minutes respectively. The odds ratios (ORs) of morning stiffness (≥60 minutes) for the presence on arthritis were 1.49 (95% confidence interval (CI) 1.001 to 2.20), 2.21 (1.33 to 3.69) and 1.55 (0.97 to 2.47) for the Leiden EA*R*C, Groningen EA*R*C and REACH respectively. The specificities ranged between 73 to 85% but the sensitivities were low (21 to 38%). The low sensitivity indicated that the majority of all patients with arthritis did not have morning stiffness for ≥60 minutes. The PPVs were 54%, 69% and 60% respectively (Table 2) and all higher than the absolute chances on arthritis without assessing morning stiffness (these were 46%, 56%, 51%). The AUCs were very low (Leiden EA*R*C 0.52, Groningen EA*R*C 0.57, REACH 0.54); therefore, no duration with optimal discriminative ability was ascertained. When morning stiffness was defined as ≥90 minutes (present in 10 to 21% of arthralgia patients), the specificity increased to 85 to 93% (Table 2). This indicated that almost all arthralgia patients without arthritis did not have morning stiffness for ≥90 minutes.

**Table 1:** **Baseline characteristics of the arthralgia patients**

	**Leiden EA** ***R*** **C**			**Groningen EA** ***R*** **C**			**REACH**		
	**All n = 807**	**No arthritis n = 435**	**Arthritis n = 372**	**All n = 481**	**No arthritis n = 214**	**Arthritis n = 267**	**All n = 353**	**No arthritis n = 172**	**Arthritis n = 181**
Age, mean ± SD, years	51.4 ± 16.3	49.6 ± 15.5	53.5 ± 17.0	51.6 ± 16.4	49.0 ± 15.3	53.7 ± 16.9	48.6 ± 14.1	46.7 ± 11.7	50.3 ± 15.9
Female, n (%)	574 (71)	345 (79)	229 (62)	311 (65)	145 (68)	166 (62)	277 (79)	149 (87)	128 (71)
Gradual onset symptoms, n (%)	499 (62)	285 (67)	214 (59)	299 (63)	140 (66)	159 (61)	183 (58)	115 (71)	68 (44)
Morning stiffness, minutes	10 (0–30)	10 (0–30)	10 (0–30)	10 (0–30)	10 (0–30)	15 (0–60)	30 (0–60)	30 (0–60)	30 (0–97)
≥30 minutes, n (%)	210 (30)	106 (28)	104 (32)	146 (36)	55 (30)	91 (40)	185 (53)	87 (52)	98 (54)
≥60 minutes, n (%)	129 (18)	60 (16)	69 (21)	88 (22)	27 (15)	61 (27)	114 (32)	45 (27)	68 (38)
≥90 minutes, n (%)	71 (10)	27 (7)	44 (14)	53 (13)	15 (8)	38 (17)	73 (21)	25 (15)	48 (27)
Tender joint count	7 (2–14)	8 (3–17)	5 (2–13)	9 (4–18)	9 (4–18)	8 (3–18)	7 (3–12)	7 (2–12)	7 (3–11)
Symptom duration, weeks	11.5 (4.0-61.0)	19.3 (6.1-122)	6.9 (2.7-21.6)	18.0 (5.6-64.3)	22.9 (6.9-74.9)	16.6 (4.6-54.9)	1.7 (0.8-3.2)	2.4 (1.2-3.6)	1.3 (0.7-2.3)
<12 weeks symptom duration, n (%)	380 (51)	162 (41)	218 (62)	165 (39)	70 (38)	95 (39)	352 (100)	171 (100)	181 (100)

Values are median (interquartile range) unless indicated otherwise. Missingness per variable as follows: gradual onset symptoms (defined as start of onset >1 week) Leiden EA*R*C n = 17, Groningen EA*R*C n = 8, REACH n = 36); morning stiffness Leiden EA*R*C n = 101, Groningen EA*R*C n = 72, REACH n = 5; tender joint count Groningen EA*R*C n = 1; symptom duration/ <12 weeks symptom duration Leiden EA*R*C n = 61, Groningen EA*R*C n = 54, REACH n = 1. Patients with missing data on morning stiffness duration did not differ significantly from patients with data on morning stiffness (data not shown). EA*R*C, Early Arthritis *Recognition* Clinic; REACH, Rotterdam Early Arthritis Cohort; SD, standard deviation.

**Table 2:** **The diagnostic value of morning stiffness (different durations) in arthralgia for the presence of arthritis**

	**OR (95% CI)** ***adjusted for age and gender***	**Sensitivity**	**Specificity**	**PPV**	**NPV**
Leiden EA*R*C					
≥30 minutes	1.24 (0.89-1.73)	31.9%	72.1%	49.5%	55.2%
≥60 minutes	1.49 (1.001-2.20)	21.2%	84.2%	53.5%	55.5%
≥90 minutes	1.98 (1.18-3.30)	13.5%	92.9%	62.0%	55.6%
Groningen EA*R*C					
≥30 minutes	1.63 (1.07-2.47)	40.3%	70.0%	62.3%	48.7%
≥60 minutes	2.21 (1.33-3.69)	27.0%	85.3%	69.3%	48.6%
≥90 minutes	2.16 (1.14-4.10)	16.8%	91.8%	71.7%	47.2%
REACH					
≥30 minutes	1.07 (0.69-1.65)	54.4%	48.2%	53.0%	50.3%
≥60 minutes	1.55 (0.97-2.47)	37.8%	72.6%	59.7%	47.9%
≥90 minutes	2.05 (1.18-3.58)	26.7%	85.1%	65.8%	48.0%

OR, odds ratio; CI, confidence interval; PPV, positive predictive value; NPV, negative predictive value; EA*R*C, Early Arthritis *Recognition* Clinic; REACH, Rotterdam Early Arthritis Cohort.

### Diagnostic value of morning stiffness in early arthritis

**Figure 2:**
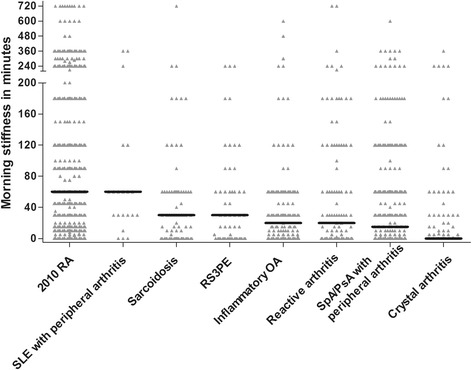
The duration of morning stiffness per diagnosis in the Leiden EAC. The black horizontal line indicates the median duration. Number of patients per diagnosis after one year of follow-up: 2010-RA n = 1,140, SLE with peripheral arthritis n = 21, sarcoidosis n = 78, RS3PE n = 60, inflammatory arthritis n = 133, reactive arthritis n = 108, SpA/PsA with peripheral arthritis n = 287, crystal arthritis n = 119. Two data points (2010-RA n = 1 and SpA/PsA with peripheral arthritis n = 1) are outside the axis limits. EAC, Early Arthritis Clinic; PsA, psoriatic arthritis; RA, rheumatoid arthritis; RS3PE, remitting seronegative symmetrical synovitis with pitting edema; SLE, systemic lupus erythematosus; SpA, spondyloarthritis.

**Figure 3:**
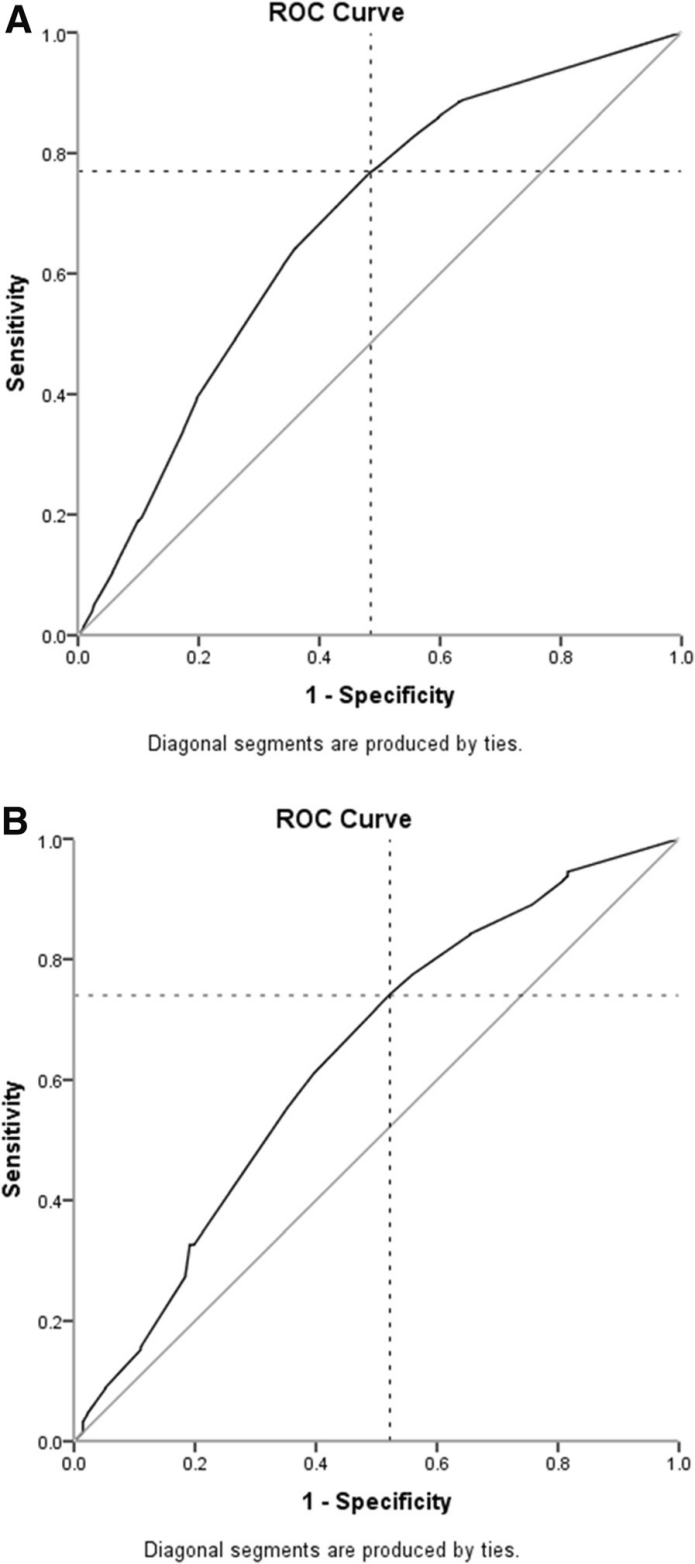
ROC curves on morning stiffness in early arthritis patients of the EAC **(A)** and ESPOIR **(B)**. The AUCs were 0.68 in the EAC and 0.64 in ESPOIR. In the EAC, the optimal cutoff point (crossing of dashed lines) reflected a sensitivity of 77% and a specificity of 52%; morning stiffness duration at this point was 27.5 minutes. When selecting the point with 80% specificity, the sensitivity was 40% and the morning stiffness duration 67.5 minutes (EAC) and a sensitivity of 33% and a morning stiffness duration 62.5 minutes (ESPOIR). AUC, area under the receiver operating characteristic curve; EAC, Early Arthritis Clinic; ESPOIR, Evaluation et Suivi de POlyarthrites Indifférenciées Récentes.

A total of 2,748 early arthritis patients were included in the EAC and 813 in ESPOIR. The median duration of morning stiffness at baseline was 30 (0 to 90) and 60 (15 to 120) minutes. Other baseline characteristics are presented in Table S1 in Additional file 1. In total, 42% and 83% of the patients were classified as 2010-RA after year one respectively. Figure 2 shows the duration of morning stiffness per diagnosis of early arthritis patients included in the EAC; the median duration in RA was 60 minutes and longer than that of other early arthritis patients (except for SLE patients). Since ESPOIR included early arthritis patients with a clinical suspicion of RA and not patients with other diagnoses, this figure was not derived for ESPOIR. Morning stiffness (≥60 minutes) was significantly associated with RA in the EAC (OR 2.92, 95% CI 2.47 to 3.44) and ESPOIR (OR 2.33, 95% CI 1.59 to 3.44), also after additional adjustments for ACPA, RF, SJC, symptom duration and ESR (OR 1.72. 95% CI 1.31 to 2.25 in EAC and OR 1.68, 95% CI 1.03 to 2.74 in ESPOIR). In both cohorts the sensitivity and specificity were around 60%. The PPV was 56.7% in EAC and 88.7% in ESPOIR, these absolute chances were a little higher than the pre-test chances on RA (42% and 83%). When morning stiffness was defined as ≥30 or ≥90 minutes consistent results were observed (Table 3). The AUCs of the presence of morning stiffness for RA in both cohorts were 0.68 (95% CI 0.66 to 0.70) and 0.64 (95% CI 0.58 to 0.69). The duration with the optimal combination of sensitivity and specificity for RA was 27.5 minutes in the EAC and 25.0 minutes in ESPOIR (Figure 3).

**Table 3:** **The diagnostic value of morning stiffness (duration and severity) in early arthritis for classifying RA**

	**OR (95% CI)** ***adjusted for age and gender***	**OR (95% CI)** ***adjusted for age, gender, SJC, ACPA, RF ESR and symptom duration***	**Sensitivity**	**Specificity**	**PPV**	**NPV**
Leiden EAC						
≥30 minutes	3.37 (2.83-4.03)	2.22 (1.66-2.96)	77.0%	51.5%	53.5%	75.5%
≥60 minutes	2.92 (2.47-3.44)	1.72 (1.31-2.25)	61.2%	66.1%	56.7%	70.1%
≥90 minutes	2.44 (2.04-2.92)	1.61 (1.19-2.18)	39.1%	80.3%	59.0%	64.5%
VAS 34-67 mm	1.87 (1.48-2.36)	2.10 (1.43-3.09)	56.5%	60.0%	49.1%	66.8%
VAS ≥68 mm	2.38 (1.89-3.00)	1.93 (1.32-2.83)	60.7%	61.8%	55.3%	66.8%
ESPOIR						
≥30 minutes	2.64 (1.81-3.87)	1.76 (1.07-2.88)	74.2%	47.8%	87.6%	27.1%
≥60 minutes	2.33 (1.59-3.44)	1.68 (1.03-2.74)	55.4%	64.7%	88.7%	22.6%
≥90 minutes	2.02 (1.28-3.20)	1.64 (0.92-2.92)	32.5%	80.9%	89.4%	19.4%
VAS 34-67 mm	2.04 (1.32-3.17)	1.93 (1.10-3.37)	61.0%	56.3%	85.7%	25.1%
VAS 68–100 mm	2.46 (1.53-3.96)	1.65 (0.88-3.11)	57.3%	63.7%	87.6%	25.1%

RA is classified according to the 2010 ACR-EULAR criteria. VAS: (0 to 100 mm). In the analyses of VAS morning stiffness three categories were formed, the reference group was a VAS 0 to 33 mm. The sensitivity, specificity, PPV and NPV of the VAS morning stiffness were calculated against this reference group. RA, rheumatoid arthritis; OR, odds ratio; CI, confidence interval; SJC, swollen joint count; ACPA, anti-citrullinated protein antibody; RF, rheumatoid factor; ESR, erythrocyte sedimentation rate; PPV, positive predictive value; NPV, negative predictive value; EAC, Early Arthritis Clinic; VAS, visual analogue scale; ESPOIR, Evaluation et Suivi de POlyarthrites Indifférenciées Récentes; ACR, American College of Rheumatology; EULAR, European League Against Rheumatism.

Both cohorts also recorded the severity of morning stiffness. In both cohorts morning stiffness duration and severity were correlated (Spearman rho 0.42, *P* <0.001 in EAC and Spearman rho 0.54, *P* <0.001 in ESPOIR) Early arthritis patients with moderate severity (VAS 34 to 67 mm) had an OR on RA of 1.87 (95% CI 1.48 to 2.36) and 2.04 (1.32 to 3.17) and the patients in the most severe category (VAS ≥68 mm) had ORs of 2.38 (95% CI 1.89 to 3.00) and 2.46 (95% CI 1.53 to 3.96) respectively. Using the VAS, the AUCs were 0.62 (95% CI 0.60 to 0.65) in EAC and 0.62 (95% CI 0.57 to 0.68) in ESPOIR. The AUCs of morning stiffness duration and morning stiffness severity were not statistically significantly different.

### Prognostic value of morning stiffness in early RA

RA patients reporting morning stiffness (≥60 minutes) at baseline did not have more severe radiographic progression (Figure S1A, B in Additional file 1) over time. During 10 years of follow-up, 23% (n = 257) of the RA patients in the EAC obtained DMARD-free sustained remission; in ESPOIR this was 10% (n = 65) during five years of follow-up. Morning stiffness (≥60 minutes) was not associated with achieving remission (Figure S1C, D in Additional file 1). Defining morning stiffness as ≥30 or ≥90 minutes or using VAS scores did not evidently change the results (Figure S1E-H in Additional file 1). In 128 EAC RA patients that achieved remission (after median 2.4 years) the VAS at inclusion and at time of remission were available; it declined from 52.5 mm at baseline (IQR 25.8 to 75.8) to 13.5 mm (IQR 1.3 to 35.8, *P* <0.005).

## Discussion

Morning stiffness is one of most commonly evaluated symptoms in the diagnostic process of joint symptoms because its presence is thought to be characteristic for arthritis or RA. Others, however, have doubted the value of morning stiffness in this respect [4,5]. The absence of large-scale studies on the diagnostic value of morning stiffness prompted us to initiate the present study. The subject is timely because of the current focus on early identification of arthritis and RA [17,21]. Furthermore, assessing the presence of or duration of morning stiffness is relatively easy, illustrating its potential utility. We aimed to determine the diagnostic and prognostic value of morning stiffness. To this end we performed a comprehensive study in different types of patients. We observed that arthralgia patients with morning stiffness for ≥60 minutes more often had arthritis than patients without morning stiffness, but the discriminative ability of this single variable (AUC) was low. Among early arthritis patients, RA patients experienced morning stiffness more frequently. Also here the discriminative ability of morning stiffness alone was moderate. This indicates that evaluation of morning stiffness is helpful in the diagnostic process in clinical practice but that it should be combined with other characteristics for optimal discrimination.

Advantageous in this study is that morning stiffness was evaluated in many arthralgia and early arthritis patients (>5,000 in total) included in several European cohorts. The arthralgia datasets had different inclusion criteria. The studied EA*R*C arthralgia patients were referred by their GPs because they doubted the presence of arthritis. The threshold for referral to the EA*R*Cs was low and the GP delays short. [13] Although the diagnostic value of morning stiffness of persons with joint symptoms in general practices was not studied, the arthralgia patients studied here represent the subset of arthralgia patients in which GPs were concerned about the presence of arthritis. Patients in REACH had to fulfil several criteria. Presence of morning stiffness was a criterion, explaining the higher median duration of morning stiffness in REACH patients. Despite the differences in patients’ selection, the test characteristics of morning stiffness for arthritis in the different arthralgia datasets were comparable, which strengthens the validity of the findings.

The early arthritis cohorts also had different inclusion criteria. ESPOIR included patients that were considered susceptible to have or develop RA, whereas the EAC included all types of early arthritis patients. Consequently, the percentage of patients fulfilling the 2010 criteria at year one and the PPV were higher in ESPOIR than in the EAC and the difference in morning stiffness between RA and non-RA patients was larger in the EAC. Nonetheless, the ORs, AUCs and test characteristics were almost similar between the cohorts, supporting the consistency of the findings.

Emery *et al*. proposed that the suspicion of RA is increased in the presence of ≥3 swollen joints, a positive squeeze test or morning stiffness ≥30 minutes. Data supporting the latter recommendation were not published before [3] but the present data support this recommendation as in early arthritis with RA as outcome, the best sensitivity-specificity combination (sensitivity 74 to 77%, specificity 48 to 52%) was present for morning stiffness enduring ≥30 minutes. However, it should be noted that the AUCs were not high and that it remains debatable whether the discriminative ability of ≥30 minutes is clearly superior to other durations. In addition, when one should aim for a specificity of 80%, to reduce false positive results, a cutoff of ≥60 minutes is required (EAC 67.5 minutes, ESPOIR 62.5 minutes, Figure 3).

The outcome in early arthritis was fulfilling the 2010-RA criteria after one year; generally diagnoses are definite then [22]. We did not intend to evaluate the predictive ability of morning stiffness for RA in undifferentiated arthritis as done previously [22,23]. We aimed to determine the discriminative ability of morning stiffness in early arthritis for RA and to this end the diagnosis had to be ascertained with as much certainty as possible.

Our findings that morning stiffness is associated with 2010-RA, independent of other characteristics (for example ACPA, RF, SJC, ESR), are in contrast with the absence of morning stiffness in the 2010 criteria [22]. This is noteworthy as the cohorts studied here were also included in the first phase of the derivation of the criteria. Several factors may play a role. First, in the derivation process patients with diagnoses other than RA or unclassified arthritis (UA) were excluded, diminishing the contrast between the groups (for comparison we observed a larger difference within the EAC than within ESPOIR). Second, the outcome was methotrexate initiation whereas we here studied fulfilling the 2010 criteria. Furthermore, morning stiffness was missing in 760 out of 3,115 patients included in phase 1 and morning stiffness was assessed differently in the different cohorts (present/absent, </≥1 hour or some categories on duration) [10]. This heterogeneity in data collection contributed to the fact that morning stiffness was voted out in phase 1 [10]. Nevertheless, the present data reveal that patients with 2010-RA more often suffer with morning stiffness than other early arthritis patients. This association is independent of the variables in the 2010 classification criteria for RA. In our view, the present data suggest that it is helpful to evaluate morning stiffness in addition to the other items of the 2010 criteria. However, we did not intend to refine the 2010 ACR/EULAR criteria for RA. For refinement of existing criteria, a different approach, involving many rheumatologists from Europe and the USA and focusing on reaching consensus, would be required.

Our study in early arthritis assessed morning stiffness in two ways: the questions related to the duration and the severity (on a VAS). It has been reported that such descriptions poorly define the experiences of patients, which encompass temporal patterns, intensities and functional disability in the early morning [24,25]. To our knowledge, no validated method is available to evaluate these items. Thus, exploration of such items may increase the diagnostic ability of morning stiffness but were not explored here.

A limitation of our data on the severity of morning stiffness is that VAS data were missing in 19% of EAC RA patients at baseline and in almost 50% of the RA patients who achieved DMARD remission at the time of remission. A previous study on 143 patients reported that morning stiffness severity is more responsive than morning stiffness duration [26]. We did not study the responsiveness of morning stiffness but noted that morning stiffness had almost disappeared when DMARD-free sustained remission was achieved. Furthermore, we also did not intend to study in detail whether the severity or the duration of morning stiffness is a better discriminator. Nonetheless, we did observe in early arthritis patients that both measures correlated moderately and that the AUC of morning stiffness severity was not superior to that of morning stiffness duration.

Another limitation of this study is that no longitudinal evaluations on the arthralgia patients were done. Recently, a prediction rule for arthritis development in ACPA-positive arthralgia patients was derived and morning stiffness ≥1 hour was part of this rule [27]. This supports the notion that including morning stiffness in the diagnostic process is valuable. More longitudinal studies on arthralgia patients are needed.

Although the circadian rhythm is associated with nightly activation of the inflammatory processes and elevation of pro-inflammatory cytokines (for example TNF-α and IL-6) [28-30], the exact mechanisms causing the stiffness in the early morning are unknown. In addition. the cortisol rhythm might contribute to the increased inflammation at night due to the low nocturnal levels of cortisol [31]. Despite the association with inflammation, morning stiffness at first presentation of RA was not associated with radiological progression or RA persistence over time. Other studies reported that morning stiffness is associated with functional disability, and work loss and therefore impacts on individual RA patients’ lives [2,11,12].

## Conclusions

The additional relevance of present data is that it provides evidence, for the first time on a large study, that incorporating morning stiffness in the diagnostic process of joint symptoms in daily practice is valuable. A duration of morning stiffness of 30 minutes had the best combination of sensitivity and specificity (74 to 77% and 48 to 52% respectively). However, in case one prefers to be specific (specificity ≥80%) a cutoff duration of 60 minutes is preferable.

## Additional file

Additional file 1:
**Overview of baseline characteristics, long-term outcomes and supplemental methods.**

## Abbreviations

ACPA: anti-citrullinated protein antibody
ACR: American College of Rheumatology
ARA: American Rheumatism Association
AUC: area under the curve
CI: confidence interval
DMARD: disease-modifying antirheumatic drug
EAC: Early Arthritis Clinic
EA*R*C: Early Arthritis *Recognition* Clinic
ESPOIR: Evaluation et Suivi de POlyarthrites Indifférenciées Récentes
ESR: erythrocyte sedimentation rate
EULAR: European League Against Rheumatism
GP: general practitioner
ICC: intraclass correlation coefficients
IL-6: interleukin 6
IQR: interquartile range
mm: millimetre
NPV: negative predictive value
OA: osteoarthritis
OR: odds ratio
PPV: positive predictive value
PsA: psoriatic arthritis
RA: rheumatoid arthritis
REACH: Rotterdam Early Arthritis Cohort
RF: rheumatoid factor
RS3PE: remitting seronegative symmetrical synovitis with pitting edema
SHS: Sharp-van der Heijde score
SJC: swollen joint count
SLE: systemic lupus erythematosus
SpA: spondyloarthritis
TNF-α: tumour necrosis factor alpha
UA: unclassified arthritis
VAS: visual analogue scale
