# Supplementary material for: Reappraisal of the diagnostic and prognostic value of morning stiffness in arthralgia and early arthritis: results from the Groningen EARC, Leiden EARC, ESPOIR, Leiden EAC and REACH
Source: Arthritis Res Ther. 2015 Apr 23;17(1):108. doi: 10.1186/s13075-015-0616-3 (PMC4445798; doi:10.1186/s13075-015-0616-3)
Supplement: Additional file 1: — Overview of baseline characteristics, long-term outcomes and supplemental methods. [file 13075_2015_616_MOESM1_ESM.docx]

`

**Additional file**

**Additional file 1: Table S1. Baseline characteristics of the early arthritis patients subdivided into 2010-RA and other**

|  | Leiden EAC | | | ESPOIR | | |
| --- | --- | --- | --- | --- | --- | --- |
|  | all early arthritis n=2748 | 2010 RA n=1140 | Other n=1608 | all early arthritis n=813 | 2010 RA n=677 | Other n=136 |
| Age, mean±SD, years | 52.1±17.1 | 55.9±15.9 | 49.4±17.4 | 48.1±12.6 | 48.6±12.3 | 45.3±13.6 |
| Female, n (%) | 1640 (60) | 365 (32) | 743 (46) | 189 (23) | 524 (77) | 100 (74) |
| Symptom duration, weeks | 14.9 (6.4-31.7) | 19.4 (10.0-36.3) | 12.0 (4.4-28.3) | 21.3 (13.2-30.9) | 21.4 (13.4-31.4) | 20.0 (12.1-28.8) |
| Swollen joints | 4 (2-9) | 8 (4-13) | 2 (1-5) | 6 (3-10) | 7 (4-11) | 3 (2-4) |
| ACPA positive, n (%) | 628 (28) | 556 (53) | 72 (6) | 316 (39) | 316 (47) | 0 |
| RF positive, n (%) | 800 (30) | 651 (57) | 149 (10) | 385 (47) | 379 (56) | 6 (4) |
| ESR mm/h | 25 (11-45) | 30 (14-50) | 19 (9-41) | 22 (12-38) | 23 (12-38) | 18 (8-36) |
| Morning stiffness, minutes | 30 (0-90) | 60 (30-120) | 15 (0-60) | 60 (15-120) | 60 (20-120) | 30 (10-60) |
| ≥30 minutes, n (%) | 1585 (61) | 848 (77) | 737 (49) | 573 (71) | 502 (74) | 71 (52) |
| ≥60 minutes, n (%) | 1189 (45) | 674 (61) | 515 (34) | 423 (52) | 375 (55) | 48 (35) |
| ≥90 minutes, n (%) | 730 (28) | 431 (39) | 299 (20) | 246 (30) | 220 (33) | 26 (19) |
| VAS-MS ≤33mm | 699 (36) | 232 (26) | 467 (44) | 231 (29) | 173 (26) | 58 (43) |
| VAS-MS 34-67mm | 613 (31) | 301 (34) | 312 (29) | 315 (39) | 270 (40) | 45 (33) |
| VAS-MS ≥68mm | 647 (33) | 358 (40) | 289 (27) | 265 (33) | 232 (34) | 33 (24) |

Median (Interquartile range) unless indicated otherwise. VAS-MS: VAS morning stiffness. Missingness per variable as follows in all early arthritis patients; Symptom duration EAC n=220 , Swollen joint (based on 66-Swollen joint count) EAC n=137;ACPA EAC n=506, ESPOIR n=1; RF EAC n=37, ESPOIR n=1; ESR EAC n=21, ESPOIR n=11, morning stiffness EAC n=127, VAS morning stiffness EAC n=532, ESPOIR n=2.

**Supplemental Methods. Questions asked on presence mornings stiffness per data-set or cohort**

|  |
| --- |
| EA*R*C Leiden and Groningen: via self-reported questionnaire |
| Do you experience stiffness when you get up in the morning?  Answers: no *or* yes. If so for how many minutes … ? |
| REACH study: via research nurse |
| Do you experience morning stiffness? If yes, for how long? |
| Leiden EAC: via research nurse |
| Do you experience in stiffness in your joints in the morning.  And if so, how long does this stiffness endures? |
| ESPOIR: via research nurse |
| Do you experience in stiffness in your joints in the morning.  And if so, how long does this stiffness endures? |


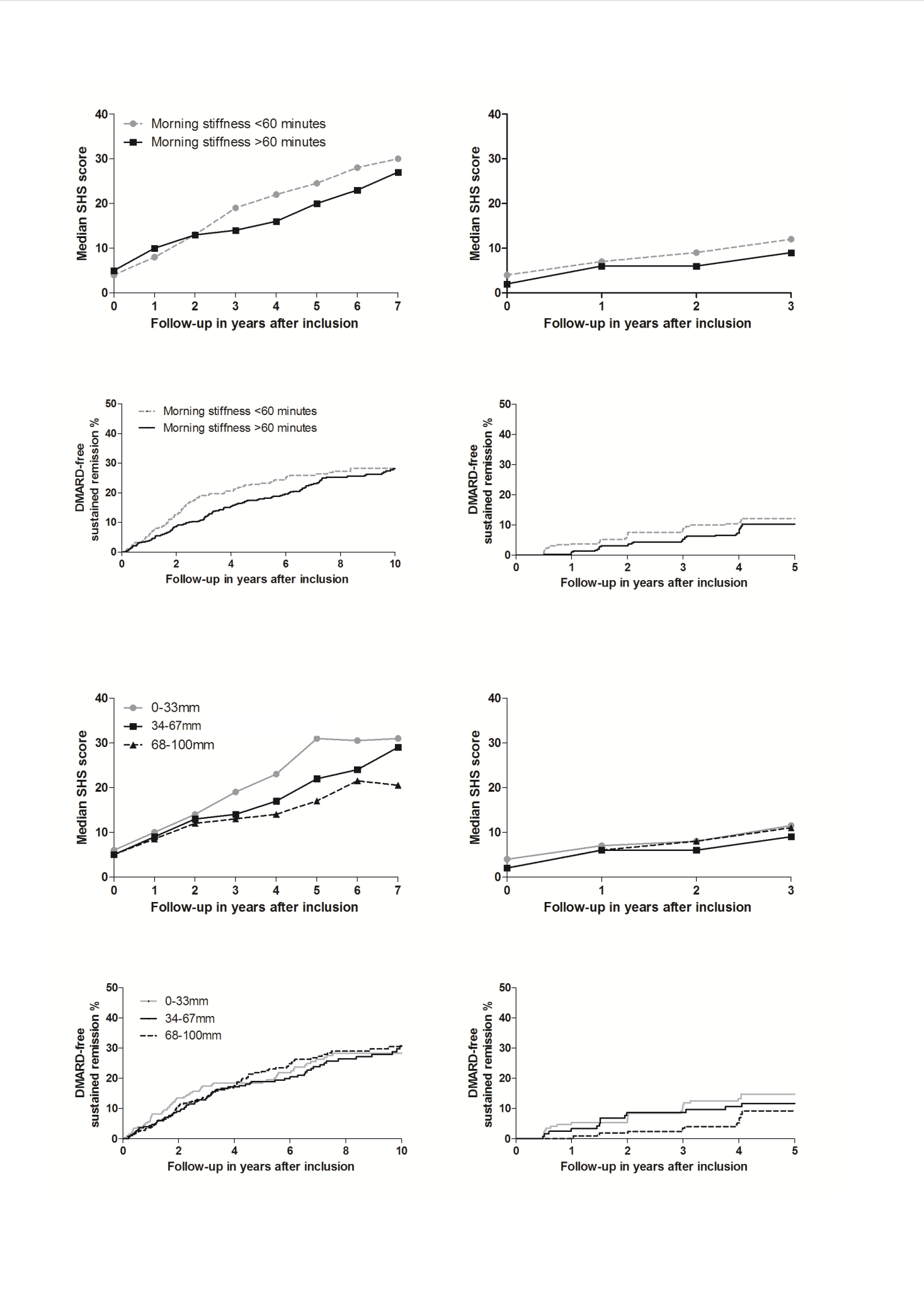
**Additional file 1: Figure S1. The severity of joint destruction (A, B, E , F) and the percentage of patients with DMARD-free sustained remission (C, D, G, H) in 2010 RA-patients in Leiden EAC (A, C, E and G) and ESPOIR (B, D, F and H) with the presence (≥60 minutes) and absence of mornings stiffness at baseline and VAS morning stiffness.**

B

A

C

D

E

F

G

H

SHS- Sharp-van der Heijde score (SHS)

**A:** β 0.98 (95%CI 0.96-1.00) p=0.054 **B:** β1.05 (95%CI 1.00-1.10) p=0.076 The beta of morning stiffness is presented with an interaction with time, indicating the relative difference in progression rate of patients with morning stiffness compared to patients without morning stiffness per year of follow-up.

**C:** EAC log rank p=0.21, HR 0.85 (95%CI 0.65-1.11). **D:** ESPOIR log rank p=0.34, HR 0.80 (95%CI 0.50-1.29).

**E:** β_34-67mm_ 1.00 (95%CI 0.97-1.03) p=0.75, β_68-100mm_ 1.01 (95%CI 0.98-1.04) p=0.48 **F:** β_34-67mm_ 1.03 (95%CI 0.96-1.10) p=0.53, β_68-100mm_ 1.02 (95%CI 0.96-1.09) p=0.45 VAS morning stiffness of 0-33mm is used as reference group.

**G:** EAC log rank p=0.919, HR_34-67mm_ 0.72 (95%CI 0.49-1.04) HR_68-100mm_ 0.79 (95%CI 0.55-1.12) VAS morning stiffness of 0-33mm is used as reference group **H:** HR_34-67mm_ 0.60 (95%CI 0.34-1.06) HR_68-100mm_ 0.52 (95%CI 0.28-0.98) VAS morning stiffness of 0-33mm is used as reference group

Performing similar analyses for the severity of joint destruction when defining morning stiffness as ≥30minutes revealed the following results: EAC β 1.00 (95%CI 0.97-1.03) p=0.98, ESPOIR β1.06 (95%CI 1.00-1.12) p=0.058. When morning stiffness was defined as ≥90minutes the results were: EAC β 0.98 (95%CI 0.96-1.00) p=0.11, ESPOIR β1.04 (95%CI 0.99-1.10) p=0.13. Sensitivity analyses on DMARD-free sustained remission were as follows with morning stiffness defined as ≥30minutes: log rank p=0.91 HR0.90 (95%CI 0.65-1.23) in EAC and log rank p=0.65 HR0.83 (95%CI 0.349-1.41) in ESPOIR and when morning stiffness was defined as ≥90minutes: log rank p=0.55 HR 0.94 (95%CI 0.71-1.23) in EAC and log rank p=0.23 HR 0.80 (95%CI 0.47-1.37) in ESPOIR.
